# Planar cell polarity protein Vangl2 interacts with M-cadherin and stabilizes its cell surface expression in mouse C2C12 myoblasts

**DOI:** 10.3389/fcell.2026.1701716

**Published:** 2026-01-16

**Authors:** Tadahiro Nagaoka, Erina Sasaki, Sakiho Takagi, Masashi Kishi, Keisuke Hitachi, Kunihiro Tsuchida

**Affiliations:** 1 Division for Therapies against Intractable Diseases, Center for Medical Science, Fujita Health University, Toyoake, Japan; 2 Division of Biological Science, Nara Institute of Science and Technology, Ikoma, Japan

**Keywords:** cell adhesion, M-cadherin, myogenesis, planar cell polarity, skeletal muscle, Vangl2

## Abstract

Skeletal muscle regeneration depends on muscle stem cells (MuSCs), in which cadherin-mediated adhesion and planar cell polarity (PCP) signaling play critical roles. M-Cadherin is the major cadherin expressed in MuSCs; however, its functional link to PCP proteins remains unclear. In this study, we demonstrate that the PCP core component Vangl2 co-localizes with M-cadherin at the MuSC-myofiber boundary and directly interacts with it in C2C12 cells. Mutagenesis analyses revealed that the catenin-binding domain of M-cadherin and the C-terminal domain of Vangl2 are required for this interaction, which uniquely enables M-cadherin to form a ternary complex with Vangl2 and β-catenin. Knockdown of Vangl2 impaired myoblast fusion, reduced the expression of MyoD and Myomixer, and decreased the cell surface stability of M- and N-cadherins, while canonical Wnt/β-catenin and Akt signaling were unaffected. These findings demonstrate that Vangl2 stabilizes cadherins at the plasma membrane and promotes myogenic differentiation, suggesting a previously unrecognized role of PCP signaling in skeletal muscle maintenance and regeneration.

## 1 Introduction

Skeletal muscle possesses a remarkable ability to regenerate after injury, largely due to the presence of skeletal muscle stem cells (MuSCs), also called satellite cells (Relaix et al., 2021). MuSCs, located between the muscle fiber membrane and the basal lamina, can activate, proliferate, and differentiate to repair damaged muscle tissue (Mukund and Subramaniam, 2020).

Cell adhesion molecules, including integrins, cadherins, and members of the IgCAM family, regulate MuSC division and differentiation (Taylor et al., 2022). Several Ca^2+^-dependent homophilic adhesion molecule cadherins, including M-, N-, and VE-cadherins, are expressed in MuSCs, and each is localized at the MuSC/myofiber boundary (Fukada et al., 2007; Goel et al., 2017). In particular, M-cadherin, a major cadherin and marker of MuSCs in skeletal muscle tissue, is associated with skeletal muscle differentiation (Donalies et al., 1991; Irintchev et al., 1994). M-cadherin gene *Cdh15-*knockout mice showed no significant muscle development or regeneration defects (Hollnagel et al., 2002), whereas M-cadherin-mediated cell adhesion regulated MuSC division (Marti et al., 2013). Furthermore, M-cadherin and N-cadherin play roles in maintaining quiescence in MuSCs (Goel et al., 2017). In addition, β-catenin, γ-catenin, and α-catenin, which are the scaffold proteins of classic cadherins and components of cadherin-based adhesion complexes, are necessary for the maintenance of MuSCs (Hung et al., 2024). Therefore, cadherin-based adhesion plays a crucial role in MuSC maintenance and stemness.

The non-canonical Wnt/planar cell polarity (PCP) pathway refers to the coordinated alignment of cellular polarity across multidimensional tissues. Six core proteins, including Celsr, Frizzled (Fzd), Dishevelled, Vangl, Prickle, and Ankrd6, are major components of the Wnt/PCP pathway (Shi, 2023). In skeletal muscle tissues, Wnt7a-Fzd7 signaling activates the Wnt/PCP pathway and stimulates the symmetric expansion of MuSCs, whereas Vangl2 knockdown disturbs Wnt7a-induced symmetric cell division of MuSCs (Le Grand et al., 2009). In addition, only Wnt7a, but not Wnt5a or Wnt3a, induces myofiber hypertrophy (von Maltzahn et al., 2011). Nitric oxide stimulates self-renewal of MuSCs by enhancing Vangl2 expression (Buono et al., 2012). The apicobasal polarity determinant Scribble associates with Vang, which is an ortholog of Vangl and acts in PCP establishment in *Drosophila* (Courbard et al., 2009). Scribble controls MuSC fate through its expression level in cells and is indispensable for muscle regeneration (Ono et al., 2015). Thus, the Wnt/PCP pathway plays a critical role in skeletal muscle maintenance and regeneration; however, its precise functions in skeletal muscle remain unclear.

Previously, we revealed that the interaction of the classic cadherins E− and N-cadherins with Vangl2 and Vangl2 induces their internalization (Nagaoka et al., 2014a; Nagaoka et al., 2014b). In the present study, we demonstrated that Vangl2 interacts with M-cadherin and promotes the stabilization of M- and N-cadherins at the cell surface. This function is mechanistically distinct from its interactions with E− and N-cadherins, highlighting its distinct functional role and providing a novel basis for understanding skeletal muscle homeostasis.

## 2 Materials and methods

### 2.1 Animals

C57BL/6J mice were obtained from Japan SLC (Hamamatsu, Japan). All animal experiments were approved by the Institutional Animal Use and Care Committee of Fujita Health University (permission number: APU19094) and were performed in accordance with the National Institutes of Health Guidelines for the Care and Use of Laboratory Animals. All methods were performed in accordance with the ARRIVE guidelines (https://arriveguidelines.org) for the reporting of animal experiments.

### 2.2 Cell lines and culture

Human embryonic kidney 293T cells were cultured in Dulbecco’s Modified Eagle Medium (DMEM) with high glucose, GlutaMAX Supplement, pyruvate (Thermo Fisher Scientific, Waltham, MA) supplemented with 10% fetal bovine serum (FBS, Cytiva, Marlborough, MA), and 10 U/mL Penicillin-Streptomycin (Thermo Fisher Scientific) in a humidified incubator at 5% CO_2_ and 37 °C. To transfect plasmid deoxyribonucleic acids (DNAs) into 293T cells, plasmids were mixed with polyethyleneimine (mixing ratio was 1 μg plasmid: 3 μL of 1 mg/mL polyethyleneimine) in OptiMEM (Thermo Fisher Scientific) and added to the culture medium without Penicillin-Streptomycin. Mouse myoblast C2C12 cells were also cultured in DMEM with high glucose, GlutaMAX Supplement, pyruvate supplemented with 10% FBS, and 10 U/mL Penicillin-Streptomycin (growth medium). To induce differentiation of C2C12 cells into myotubes, the medium was replaced with DMEM with high glucose, GlutaMAX Supplement, and pyruvate supplemented with 2% horse serum (Thermo Fisher Scientific; product # 26050088) (differentiation medium).

### 2.3 Antibodies, plasmid deoxyribonucleic acids (DNAs) and dicer-substrate short interfering ribonucleic acids (DsiRNAs)

Antibodies used in this study are listed in Supplementary Table S1. Mouse M-cadherin gene (*Cdh15*, GenBank accession number: NM_007662) was amplified from mouse cerebellum complementary DNA (cDNA) by polymerase chain reaction (PCR) and cloned into TA cloning vector pGEM-T easy (Promega, Madison, WI). M-cadherin gene, which was isolated from pGEM-T easy-M-cadherin by digestion with HindIII and EcoRI, and 3xmyc-tag oligonucleotides were ligated with pcDNA3.1+ vector digested with HindIII and XbaI using Ligation High (Toyobo, Osaka, Japan) to construct the M-cadherin-myc mammalian expression plasmid. To remove the juxtamembrane domain core region (JMDC) or catenin-binding domain (CBD) and to substitute serine residues 745, 751, 752, 755, and 756 with alanine or aspartate, PCR fragments outwardly with the mutated primers for M-cadherin were self-fused using in-fusion HD (Takara Bio, Kusatsu, Japan). The mouse β-catenin gene (GenBank accession number: NM_001165902) amplified by PCR followed by NheI and BamHI digestion and 3x hemagglutinin (HA) -tag oligonucleotides were ligated with pcDNA3.1+ vector (Thermo Fisher Scientific) digested by NheI and NotI. The human *VANGL2* gene (GenBank accession number: NM_020335) amplified from neuroblastoma SH-SY5Y cDNA library by PCR followed by HindIII and XbaI digestion was ligated with pcDNA3.1+ vector digested by HindIII and XbaI. To construct C-terminal domain deleted (ΔCT) human VANGL2 expression plasmids, PCR fragments outwardly with the mutated primers for human *VANGL2* were self-fused using in-fusion HD. All gene sequences were confirmed using ABI 3500 Genetic Analyzer (Thermo Fisher Scientific). The PCR primers used to construct the plasmids are listed in Supplementary Table S2. DsiRNAs were purchased from Integrated DNA Technologies (Coralville, IA, United States), and their sequences are shown in Supplementary Table S3.

### 2.4 Whole-mount immunofluorescence of mouse plantaris muscle

We used the method previously described by Kurosawa et al. with slight modification (Kurosawa et al., 2022). In brief, the plantaris muscles were obtained from 3-week-old female mice and immediately fixed with phosphate-buffered saline (PBS) containing 4% paraformaldehyde (PFA) for 30 min on ice, followed by three washes with PBS. The fixed muscle was blocked with PBS containing 10% FBS and 1% Triton X-100 overnight at 4 °C, and the blocking buffer was then replaced by fresh blocking buffer containing primary antibodies and incubated overnight at 4 °C. The primary antibodies were then removed, and the plantaris was washed with PBS three times. Final wash buffer was replaced by fresh blocking buffer containing fluorescent secondary antibodies and incubated overnight at 4 °C followed by three washes with PBS. The tissue was mounted on a glass slide using Fluorescence Mounting Medium (Agilent, Santa Clara, CA, United States) to avoid photobleaching. Fluorescence images were obtained using a confocal laser scanning microscope LSM-710 (Zeiss, Jena, Germany). The fluorescence signal intensity was analyzed using the Plot Profile of ImageJ (https://imagej.net/software/fiji/).

### 2.5 Immunoprecipitation and Western blotting

293T cells were lysed using lysis buffer consisting of 1% NP-40, 10% glycerol, 1 mM ethylenediaminetetraacetic acid, 0.1 mM phenylmethylsulfonylfluoride, 150 mM NaCl, and 50 mM Tris-HCl (pH 7.5), while C2C12 cells were lysed using radioimmunoprecipitation assay (RIPA) buffer consisting of 0.1% sodium dodecyl sulfate (SDS), 150 mM NaCl, and 50 mM Tris-HCl (pH 7.5). The antibodies are added into the supernatant of cell lysate and incubated with rotation at 4 °C overnight. Subsequently, 10 μL of Protein G Mag Sepharose Xtra (Cytiva) was added and further incubated for 1 h with rotation at 4 °C. Protein G beads were washed four times with lysis buffer or RIPA buffer. Protein G beads were suspended into SDS sample buffer and boiled for 5 min to denature proteins. Denatured protein samples were separated by SDS polyacrylamide gel electrophoresis (SDS-PAGE). The proteins were then transferred from the gel to polyvinylidenedifluoride (PVDF) membrane (Immobilon-P; Millipore, Burlington, MA). The membrane was blocked with Tris-buffered saline containing 0.1% Tween-20 (TBS-T) and 5% skim milk for 1 h at room temperature, followed by washing thrice with TBS-T. PVDF membrane was incubated with primary antibody diluted in Can Get Signal Solution 1 (Toyobo) overnight at 4 °C. After three washes with TBS-T, the membrane was incubated with horseradish peroxidase (HRP)-conjugated secondary antibody diluted in Can Get Signal Solution 2 (Toyobo) for 1 h at room temperature. The membrane was washed four times with TBS-T. The membrane was treated with Pierce ECL plus Western blot substrate (Thermo Fisher Scientific), and chemiluminescence images were obtained using a cooled charge-coupled device camera system (Light-Capture; ATTO, Tokyo, Japan).

### 2.6 Differentiation of C2C12 into myotube

DsiRNA was transfected into 3 × 10^3^ of C2C12 cells in a 96-well cell culture plate (Corning), and the cells were incubated for 48 h in growth medium. The medium was then replaced with differentiation medium and incubated for 7 days. The medium was replaced with fresh medium every 2 days. After differentiation, the cells were immuno-stained by anti-myosin heavy chain (MyHC) antibody, and nuclei were stained by 4′,6-diamidino-2-phenylindole (DAPI). Fluorescent images were captured using an all-in-one fluorescent microscope BZ-X800 (Keyence, Osaka, Japan) with a 10x objective lens. To determine MyHC-positive (myotubes) and DAPI-positive (nuclei) areas, the Weka Trainable Segmentation Macro in ImageJ software was used (Arganda-Carreras et al., 2017). Binary images of DAPI were processed watershed and analyzed to count the nuclei. The fusion index was calculated as the ratio of nuclei inside the MyHC-positive area in a region of interest. The number of nuclei within the MyHC-positive area was calculated by subtracting (1) the number of nuclei outside the MyHC-positive area and (2) the number of nuclei within the MyHC-positive areas that contained only one or two nuclei from the total number of nuclei (Hinkle et al., 2021).

### 2.7 Quantitative PCR (qPCR)

Total RNA was isolated from C2C12 cells using RNAiso Plus (Takara Bio). Isolated total RNA was incubated for 5 min at 65 °C and immediately cooled down on ice to denature. To obtain cDNA as a template for qPCR, reverse transcription was carried out using the ReverTra Ace qPCR RT Master Mix with gDNA Remover (Toyobo). TB Green Premix Ex Taq (Takara Bio) was used for qPCR, and reaction control and data processing were performed using Thermal Cycler Dice Real Time System TP800 (Takara Bio). Relative gene expression was quantified using the ΔΔCt method. The ribosomal protein lateral stalk subunit P0 (Rplp0) gene *36B4* expression was used as a reference. Primer sequences are listed in Supplementary Table S4.

### 2.8 Biotinylation of the cell surface proteins

To knockdown the expression of *Vangl2*, 50 nM DsiRNA was transfected into 5 × 10^4^ of C2C12 cells in a 12-well cell culture plate (Corning, Corning, NY) using the standard reverse transfection protocol of Lipofectamine RNAi max (Thermo Fisher Scientific). After 24 h, 0.5 μg of human VANGL2 expression plasmid was transfected into each well using Fugene HD (Promega) to rescue knocked-down *Vangl2* expression. Further 24 h of incubation with the growth medium, the cells were washed three times with ice-cold PBS. The wash buffer was replaced with ice-cold PBS containing 0.2 mg/mL Pierce EZ-Link NHS-PEG_4_-Biotin (Thermo Fisher Scientific) and incubated for 30 min on ice. To quench the crosslinking reaction, ice-cold TBS was added to each well. The cells were washed thrice with ice-cold TBS, followed by cell lysis using lysis buffer. Streptavidin-agarose (5 μL; Thermo Fisher Scientific) was added to the cell lysate and incubated for 1 h at 4 °C with rotation to pull-down the biotinylated proteins. Agarose beads were washed three times with lysis buffer and suspended into SDS sample buffer. The samples were then separated by SDS-PAGE and transferred onto PVDF membranes. Total biotinylated proteins were detected using peroxidase-labelled streptavidin (KPL; Gaithersburg, MD, United States).

### 2.9 Statistical analysis

All data analyses were carried out using GraphPad Prism 10 software (GraphPad software, Boston, MA). Mean (dotted line) ± standard deviation (solid line) are shown in each graph. One-way analysis of variance (ANOVA) followed by Tukey’s multiple comparison test was used to evaluate the statistical significance among three or more different conditions. Welch’s *t*-test was used to evaluate the statistical significance between two different conditions. Statistical significance was set at P < 0.05.

## 3 Results

### 3.1 M-cadherin is associated with Vangl2 in MuSCs and C2C12 cells

**FIGURE 1:**
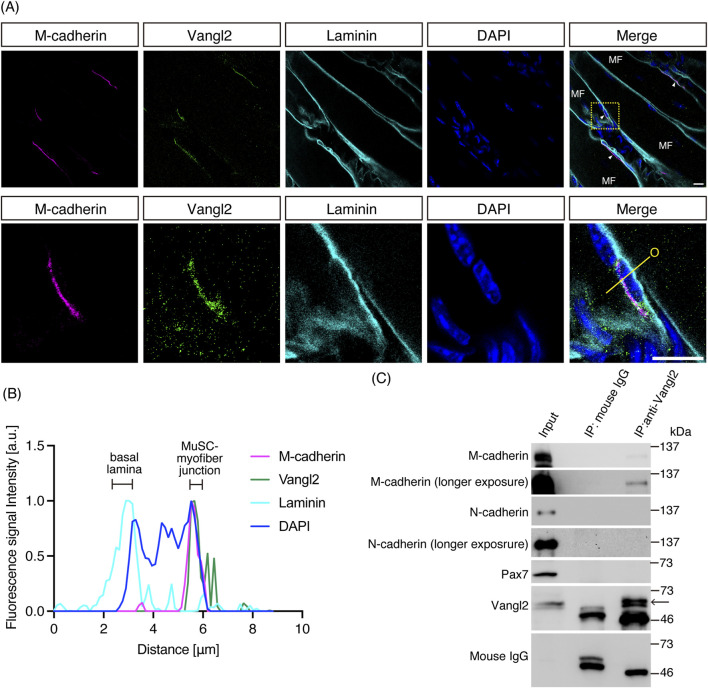
M-cadherin interacts with Vangl2 **(A)** M-cadherin and Vangl2 were co-localized in mouse plantaris muscle. The area delineated by the yellow dashed box in the upper panel is presented at higher magnification in the lower panels. MuSCs are marked by white arrowheads, and the muscle fiber is denoted as “MF.” **(B)** Fluorescence signal intensity along the yellow line in the merged image of **(A)**. “O” indicates origin of the yellow line. Scale bar: 10 μm **(C)** Immunoprecipitation of C2C12 cell lysate by anti-Vangl2 antibody. The arrow indicates the band of Vangl2 proteins.

**FIGURE 2:**
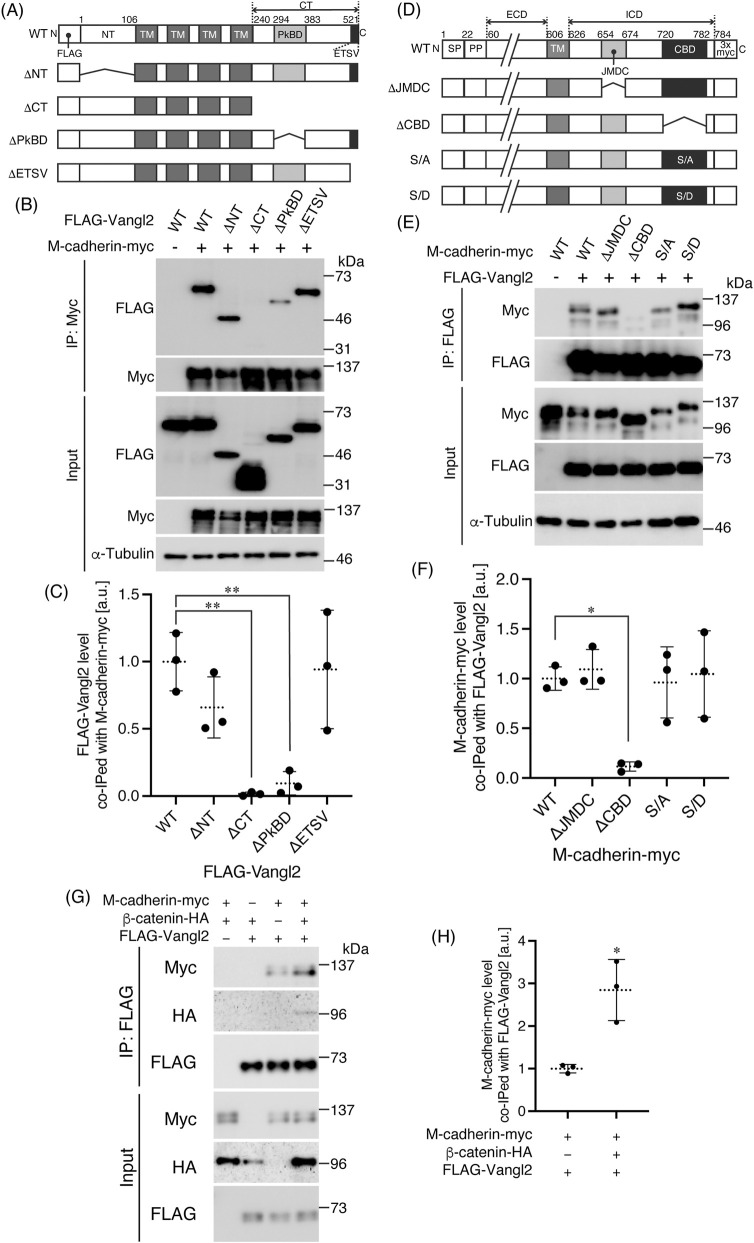
Catenin binding domain of M-cadherin interacts with C-terminal domain of Vangl2 **(A)** Schematic representation of the FLAG-Vangl2 mutant constructs. TM: transmembrane domain **(B,C)** M-cadherin-myc WT and FLAG-Vangl2 mutants were co-expressed in 293T cells, and their lysates were immunoprecipitated by anti-myc antibodies. Band intensity of co-immunoprecipitated FLAG-Vangl2 mutants relative to the intensity of immunoprecipitated M-cadherin-myc was measured by ImageJ and plotted in **(C)** (n = 3). One-way ANOVA: P = 0.0012, Tukey’s multiple comparisons test: **; P < 0.005. **(D)** Schematic representation of the M-cadherin-myc mutant constructs. SP: signal peptide, PP: pro-peptide, ECD: extracellular domain, ICD: intracellular domain. **(E,F)** FLAG-Vangl2 WT and M-cadherin mutants were co-expressed in 293T cells, and their lysates were immunoprecipitated by anti-FLAG antibodies. Band intensity of co-immunoprecipitated M-cadherin-myc mutants relative to the intensity of immunoprecipitated FLAG-Vangl2 was measured by ImageJ and plotted in **(F)** (n = 3). One-way ANOVA; P = 0.0067, Tukey’s multiple comparisons test: *; P < 0.05. **(G,H)** β-catenin-HA was co-expressed with M-cadherin and Vangl2 in 293T cells. Cell lysate was immunoprecipitated by anti-FLAG antibodies. Band intensity of co-immunoprecipitated M-cadherin-myc relative to the intensity of immunoprecipitated FLAG-Vangl2 was plotted in **(H)** (n = 3). Welch’s *t*-test: *; P < 0.05.

To observe the localization of M-cadherin and Vangl2 in the skeletal muscle tissue, we performed whole-mount immunofluorescence using 3-week-old mouse plantaris muscles (Figure 1A). We chose the plantaris of 3-week-old mice because the tissue is sufficiently thin to prepare whole-mount immunofluorescence specimens on slides, and because the density of MuSCs is high during this developmental stage. M-cadherin was expressed at the junction of the muscle fibers of MuSCs, and M-cadherin fluorescent signal was almost concordant with that of Vangl2 (Figure 1B). Vangl2 signal was slightly broader than M-cadherin signal, however. Meanwhile, laminins, which are basal lamina proteins, were inconsistently localized with Vangl2 (Figure 1B). Therefore M-cadherin and Vangl2 co-localized at the junction of the muscle fibers of MuSCs. To explore the physical interaction between M-cadherin and Vangl2, we performed a co-immunoprecipitation assay. Mouse myoblast C2C12 cell lysates were immunoprecipitated with anti-Vangl2 antibodies. M-cadherin was co-immunoprecipitated with Vangl2 (Figure 1C). C2C12 cells express not only M-cadherin but also N-cadherin. However, unlike in neurons, we did not detect N-cadherin bands in this co-immunoprecipitation assay (Nagaoka et al., 2014b). To determine M-cadherin binding site within Vangl2, we transfected M-cadherin-myc and Vangl2 mutants (Figure 2A; Nagaoka et al., 2014b) into 293T cells and immunoprecipitated their lysates with anti-myc antibodies. N-terminal domain (ΔNT) and PDZ binding motif (ΔETSV), which are the last four amino acid residues at the C-terminus, were deleted, and Vangl2 mutants continued to interact with M-cadherin-myc (Figures 2B,C; ΔNT 66% ± 23%, ΔETSV 94% ± 44% vs. wildtype (WT)) (Dreyer et al., 2022). On the other hand, the interaction between Vangl2 ΔCT and Prickle binding domain deleted (ΔPkBD) mutants and M-cadherin-myc was significantly reduced (Figures 2B,C; ΔCT 1.5% ± 1.4%, ΔPkBD 9.5% ± 8.6% vs. WT). Thus, the C-terminal domain of Vangl2 is required for binding to M-cadherin, which is consistent with E− and N-cadherins (Nagaoka et al., 2014a; Nagaoka et al., 2014b). To determine Vangl2 binding site within M-cadherin, we constructed M-cadherin-myc harboring in-frame deletions or point mutations in the intracellular domain (ICD) (Figure 2D). These constructs were co-expressed with FLAG-Vangl2 in 293T cells, and cell lysates were immunoprecipitated with anti-FLAG antibodies. JMDC of E-cadherin interacts with p120 catenin and regulates cell adhesion (Ishiyama et al., 2010). This motif is highly conserved among M-cadherins. JMDC deleted mutant (ΔJMDC) continued to interact with Vangl2 (Figures 2E,F; 110% ± 20% vs. WT). Thus, JMDC of M-cadherin is dispensable for its interaction with Vangl2. As the other domain responsible for CBD adhesion interaction with the scaffold proteins β-catenin and plakoglobin, which are linked to the actin cytoskeleton via α-catenin (Conacci-Sorrell et al., 2002). CBD deleted mutant (ΔCBD) showed significantly reduced interaction with Vangl2 (Figures 2E,F; 12% ± 5% vs. WT). This suggests that, similar to E− and N-cadherins, the CBD of M-cadherin is responsible for binding to Vangl2 (Nagaoka et al., 2014a; Nagaoka et al., 2014b). Phosphorylation of serine residues in the CBD of E-cadherin is required to interact with β-catenin (McEwen et al., 2014). Furthermore, it has been suggested that the phosphorylation of serine residues in the CBD of E− and N-cadherins is required for binding to Vangl2 (Nagaoka et al., 2014a; Nagaoka et al., 2014b). Interestingly, M-cadherin mutant in which five serine residues within the CBD were substituted with alanine (S/A) to mimic a dephosphorylated state retained its ability to bind Vangl2 (Figures 2E,F; 96% ± 36% vs. WT). When these serine residues were substituted with aspartate (S/D) to mimic phosphorylated state, this mutant was still capable of interacting with Vangl2 (Figures 2E,F; 100% ± 40% vs. WT). We confirmed that both ΔCBD and S/A mutants were not co-immunoprecipitated with endogenous β-catenin in 293T cells, and S/D mutant significantly decreased affinity to β-catenin compared with M-cadherin WT but still continued to interact with β-catenin (Supplementary Figure S1A,B; ΔCBD: 4.0% ± 2.0%, S/A: 3.8% ± 0.5%, S/D: 40% ± 16%, vs. WT). Furthermore, M-cadherin-myc, FLAG-Vangl2, and β-catenin-HA were co-expressed in 293T cells, and the lysates were immunoprecipitated using anti-FLAG antibodies. Co-immunoprecipitated M-cadherin band intensity of M-cadherin-myc, FLAG-Vangl2, and β-catenin-HA co-expressed lysate was 2.8 ± 0.7 times higher than that of M-cadherin-myc and FLAG-Vangl2 co-expressed lysate (Figures 2G,H). Moreover, β-catenin-HA was also co-immunoprecipitated when co-expressed with both M-cadherin-myc and FLAG-Vangl2; however, β-catenin-HA was not co-immunoprecipitated when co-expressed with Vangl2 only (Figure 2G). Reciprocally, immunoprecipitation with anti-myc antibodies showed that the amount of co-immunoprecipitated FLAG-Vangl2 in lysates co-expressing all three proteins was 2.9 ± 0.8-fold higher than that in lysates co-expressing M-cadherin-myc and FLAG-Vangl2 (Supplementary Figure S1C,D). While the level of co-immunoprecipitated β-catenin-HA was unchanged regardless of FLAG-Vangl2 expression (Supplementary Figure S1E). Although β-catenin and Vangl2 compete interaction with N-cadherin and E-cadherin (Nagaoka et al., 2014a; Nagaoka et al., 2014b), β-catenin and Vangl2 may make complex with M-cadherin. In addition, the structure of the cadherin ICD/Vangl2 CT complex was predicted using AlphaFold3 (Abramson et al., 2024). The predicted structure showed that the C-terminal region in β-catenin binding domain of E-cadherin, N-cadherin and M-cadherin contacts with the upstream region of Prickle binding domain of Vangl2 (Supplementary Figure S2A). These predictions are consistent with our results. These results suggested that the interaction between M-cadherin and Vangl2 was slightly different from that between E− and N-cadherin.

### 3.2 Vangl2 is required for myotube differentiation of C2C12 cells

**FIGURE 3:**
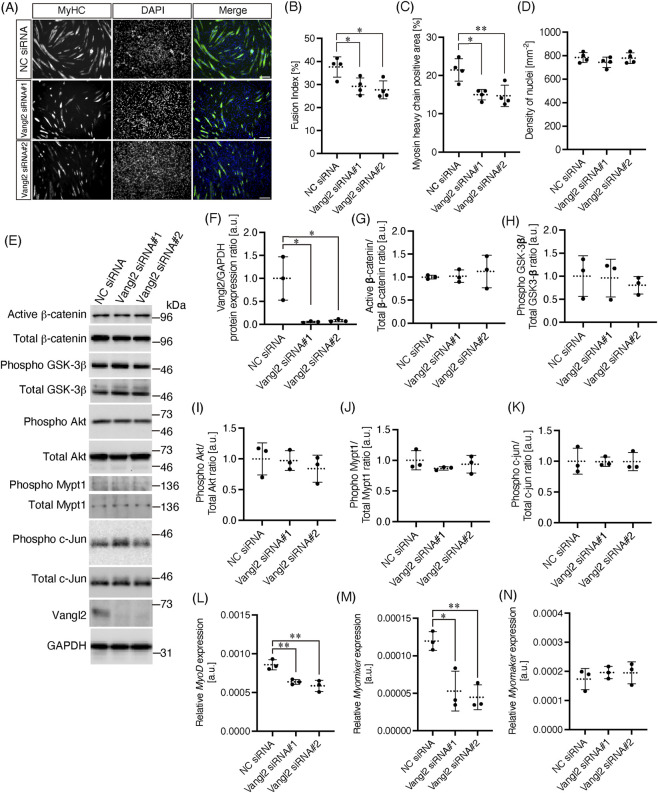
Vangl2 is required for myotube differentiation of C2C12 cells **(A)** C2C12 cells transfected with DsiRNAs were treated with 2% horse serum to induce myotube differentiation and incubated for 7 days. Cells were immune stained by MyHC. Scale bar: 200 μm. **(B)** Fusion index was calculated from C2C12 cells differentiated for 7 days (n = 4). One-way ANOVA: P = 0.014. **(C)** To calculate fusion index, MyHC-positive area was measured (n = 4). One-way ANOVA: P = 0.0057. **(D)** To calculate fusion index, number of nuclei was counted, and density of nuclei was plotted (n = 4). One-way ANOVA: P = 0.39. **(E)** C2C12 cells transfected with DsiRNAs were incubated in growth medium for 2 days and then lysed. Phosphorylation levels of canonical Wnt or non-canonical Wnt/PCP signaling related components were evaluated by Western blotting. **(F)** Band intensity of Vangl2 relative to that of GAPDH was plotted (n = 3). One-way ANOVA: P = 0.0085. **(G)** Band intensity of active β-catenin relative to that of total β-catenin was plotted (n = 3). One-way ANOVA: P = 0.77. **(H)** Band intensity of phospho GSK-3β (Ser9) relative to that of total GSK-3β was plotted (n = 3). One-way ANOVA: P = 0.79. **(I)** Band intensity of phospho Akt (Ser473) standardized by total Akt was plotted (n = 3). One-way ANOVA: P = 0.65. **(J)** Band intensity of phospho Mypt1 (Thr853) standardized by total Mypt1 was plotted (n = 3). One-way ANOVA: P = 0.86. **(K)** Band intensity of phospho c-Jun (Ser63) standardized by total c-Jun was plotted (n = 3). One-way ANOVA: P = 1.0. **(L–N)** Gene expression levels of *MyoD*, *Myomixer*, and *Myomaker* were measured in C2C12 cells treated with DsiRNAs for 2 days. One-way ANOVA: *MyoD*; P = 0.0032, *Myomixer*; P = 0.0062, *Myomaker*; P = 0.66. Tukey’s multiple comparisons test: *; P < 0.05, **; P < 0.005.

To explore the physiological functions of the interaction between M-cadherin and Vangl2, we knocked down the expression of *Vangl2* using DsiRNAs and induced differentiation of the cells into myotubes. Differentiation was induced by 2% horse serum and differentiated cells were immunostained with an anti-MyHC antibody (Figure 3A). The fusion index is a quantitative measure in skeletal muscle research because it provides a simple and reliable assessment of myoblast differentiation and myotube formation. This differentiation and quantification system is widely used (Hitachi et al., 2019; Chakraborty et al., 2022). The fusion index was significantly decreased following transfection with Vangl2 DsiRNAs (Figure 3B: NC siRNA; 37.6% ± 4.4%, Vangl2 siRNA#1; 29.2% ± 3.7%, Vangl2 siRNA#2; 27.7% ± 3.9%). Simply, MyHC-positive area was also decreased after Vangl2 DsiRNAs transfection (Figure 3C: NC siRNA; 21.4% ± 2.9%, Vangl2 siRNA#1; 15.0% ± 1.4%, Vangl2 siRNA#2; 14.7% ± 2.8%). Low cell density reduces cell–cell contact and suppresses differentiation of C2C12 cells (Tanaka et al., 2011). Therefore, we measured the cell density by counting nuclei and observed no significant difference in cell density under all conditions (Figure 3D: NC siRNA; 785 ± 42 mm^−2^, Vangl2 siRNA#1; 744 ± 44 mm^-2^, Vangl2 siRNA#2; 780 ± 46 mm^−2^). M-cadherin-mediated signaling attenuates phosphorylation of β-catenin through glycogen synthase kinase-3 beta (GSK-3β), and this effect induces myogenic differentiation (Wang et al., 2013). We investigated whether Vangl2 affects β-catenin phosphorylation using antibodies specific for the active (dephosphorylated) form of β-catenin. *Vangl2* was efficiently knocked down by DsiRNAs in undifferentiated C2C12 cells (Figures 3E,F: NC siRNA, 100% ± 47%; *Vangl2* siRNA#1, 5.5% ± 1.7%; *Vangl2* siRNA#2, 8.4% ± 2.8%). Despite this robust knockdown, none of the signaling pathways typically associated with β-catenin function showed detectable changes in activation. Specifically, the levels of active β-catenin (Figures 3E,G: NC siRNA, 100% ± 4%; *Vangl2* siRNA#1, 102% ± 14%; *Vangl2* siRNA#2, 112% ± 35%), phosphorylated GSK-3β (Figures 3E,H: NC siRNA, 100% ± 44%; *Vangl2* siRNA#1, 96.1% ± 41.1%; *Vangl2* siRNA#2, 80.3% ± 19.0%), and phosphorylated Akt (Laprise et al., 2004; Gardner et al., 2012, Figures 3E,I: NC siRNA, 100% ± 26%; *Vangl2* siRNA#1, 97.3% ± 16.1%; *Vangl2* siRNA#2, 84.1% ± 22.0%) remained unchanged. Likewise, downstream targets of the Rho/ROCK and JNK pathways which are related to Wnt/PCP pathway—Mypt1 and c-Jun, respectively—also showed no differences in phosphorylation (Hayes et al., 2018; Zhang et al., 2018, Figures 3E,J,K: Mypt1, NC siRNA, 100% ± 15%; *Vangl2* siRNA#1, 86.8% ± 2.7%; *Vangl2* siRNA#2, 93.6% ± 14.4%. c-Jun, NC siRNA, 100% ± 21%; *Vangl2* siRNA#1, 99.2% ± 7.6%; *Vangl2* siRNA#2, 99.4% ± 14.9%). Thus, although many pathways were assessed, *Vangl2* knockdown did not alter any of the expected upstream signaling nodes. In contrast to the absence of signaling changes, gene expression analysis revealed specific transcriptional differences. *Vangl2* knockdown significantly reduced *MyoD* expression (NC siRNA, (8.6 ± 0.7) × 10^–4^ a. u.; *Vangl2* siRNA#1, (6.4 ± 0.3) × 10^–4^ a. u.; *Vangl2* siRNA#2, (5.9 ± 0.7) × 10^–4^ a. u.) and *Myomixer* expression (NC siRNA, (1.2 ± 0.1) × 10^–4^ a. u.; *Vangl2* siRNA#1, (5.3 ± 2.7) × 10^–5^ a. u.; *Vangl2* siRNA#2, (4.5 ± 1.7) × 10^–5^ a. u.) (Figures 3L,M), whereas *Myomaker* levels were unaffected (Figure 3N). Other myogenic genes, including *Pax7*, *Myf5*, *Myogenin*, and the M-cadherin gene *Cdh15*, were also unchanged (Supplementary Figure S3). Together, these results clarify that although Vangl2 loss does not influence canonical signaling pathways, it selectively regulates *MyoD* and *Myomixer* expression, providing a likely mechanism for its role in myoblast fusion (Zhang et al., 2020).

### 3.3 Vangl2 stabilizes membrane expression of M-cadherin in undifferentiated C2C12 cells

**FIGURE 4:**
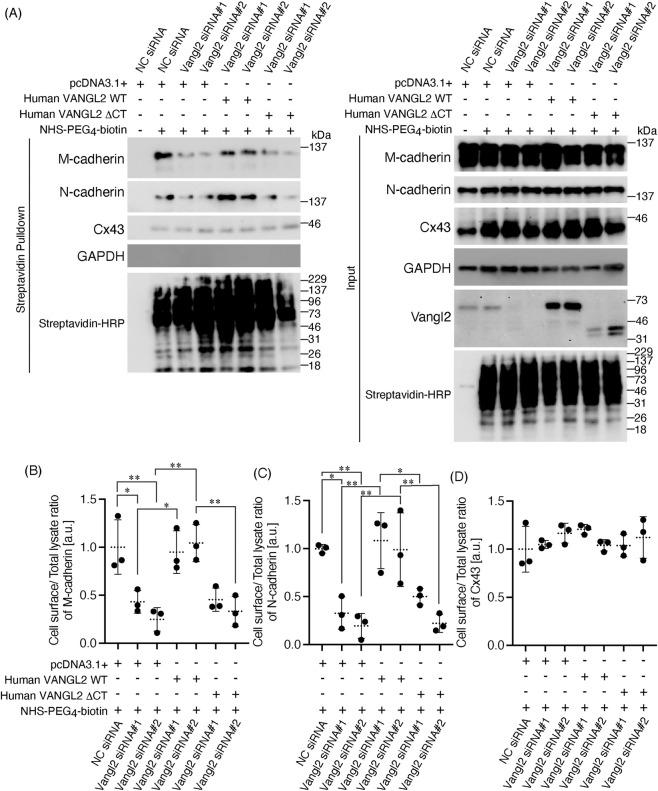
Vangl2 knockdown reduced cell surface expression of M-cadherin in C2C12 cells **(A)** C2C12 cells transfected with DsiRNAs were incubated in growth medium for 2 days and then treated with NHS-PEG_4_-Biotin on ice to label cell surface proteins with biotin. Biotinylated proteins were pulled down by streptavidin-agarose gel and detected by Western blotting. **(B)** Cell surface M-cadherin expression level was measured and plotted (n = 3). One-way ANOVA: P = 0.0002. **(C)** Cell surface N-cadherin expression level was measured and plotted (n = 3). One-way ANOVA: P = 0.0002. **(D)** Cell surface Cx43 expression level was measured and plotted (n = 3). One-way ANOVA: P = 0.51. Tukey’s multiple comparisons test: *; P < 0.05, **; P < 0.005.

We were unable to identify the specific signaling pathway in which the interaction between M-cadherin and Vangl2 plays a functional role. Therefore, we investigated whether this interaction also has physical effects, such as promotion of N-cadherin endocytosis by Vangl2 as previously reported (Nagaoka et al., 2014a). C2C12 cells transfected with DsiRNAs were incubated in growth medium for 48 h and then cooled on ice to arrest membrane trafficking. Subsequently, the proteins exposed outside the cell membrane were labeled with biotin. Biotinylated proteins were analyzed by streptavidin pull-down following Western blotting to measure the cell surface expression levels. The cell surface expression of M-cadherin was reduced when Vangl2 was knocked down compared with NC siRNA transfected cells (Figures 4A,B: NC siRNA; 100% ± 28%, *Vangl2* siRNA#1; 43.0% ± 1.7%, *Vangl2* siRNA#2; 24.8% ± 12.3%). To rescue the *Vangl2* expression knocked-down by DsiRNAs, cells were transfected with human VANGL2 expression plasmids. When human VANGL2 WT was additionally transfected, surface expression of M-cadherin was increased, however, additional VANGL2 ΔCT transfection did not affect surface expression of M-cadherin (*Vangl2* siRNA#1 + VANGL2 WT; 94.8% ± 22.1%, *Vangl2* siRNA#2 + VANGL2 WT; 104% ± 19%, *Vangl2* siRNA#1 + VANGL2 ΔCT; 45.3% ± 12.2%, *Vangl2* siRNA#2 + VANGL2 ΔCT; 33.1% ± 15.7%). Similarly, the surface expression of N-cadherin was reduced following transfection with *Vangl2* DsiRNAs (Figures 4A,C: NC siRNA; 100% ± 4%, *Vangl2* siRNA#1; 32.8% ± 17.0%, *Vangl2* siRNA#2; 19.5% ± 12.9%). In addition, human Vangl2 WT expression rescued surface expression, whereas Vangl2 ΔCT could not rescue (Figures 4A,D: *Vangl*2 siRNA#1 + VANGL2 WT; 109% ± 29%, *Vangl2* siRNA#2 + VANGL2 WT; 99.0% ± 38.4%, *Vangl2* siRNA#1 + VANGL2 ΔCT; 50.1% ± 8.6%, *Vangl2* siRNA#2 + VANGL2 ΔCT; 22.3% ± 9.4%). In contrast, cell surface expression of gap junction protein Connexin 43 (Cx43) which plays important role in myoblast fusion did not alter following *Vangl2* DsiRNAs transfection (Araya et al., 2005; Figures 4A,D: NC siRNA; 100% ± 24%, *Vangl2* siRNA#1; 105% ± 4%, *Vangl2* siRNA#2; 117% ± 10%, *Vangl*2 siRNA#1 + VANGL2 WT; 121% ± 5%, *Vangl2* siRNA#2 + VANGL2 WT; 104% ± 6%, *Vangl2* siRNA#1 + VANGL2 ΔCT; 104% ± 12%, *Vangl2* siRNA#2 + VANGL2 ΔCT; 112% ± 22%). In addition, biotinylation of the cytoplasmic protein glyceraldehyde-3-phosphate dehydrogenase (GAPDH) was not detected. This indicated that the biotin-labeling reagent did not penetrate the plasma membrane. These results suggested that Vangl2 stabilized M-cadherin and N-cadherin on the surface of C2C12 cells. This is consistent with the requirement for N- and M-cadherin recruitment to cell–cell contacts, and their stabilization at the plasma membrane during myogenic differentiation of C2C12 cells (Charrasse et al., 2013; Ozawa, 2015).

## 4 Discussion

Cadherin-dependent cell adhesion controls the quiescence and maintenance of MuSCs (Goel et al., 2017; Hung et al., 2024). Here, we show a novel possible interaction between M-cadherin and Vangl2 in MuSCs and C2C12 cells.

A previous study described that Vangl2 expression was not detected in quiescent MuSCs but was upregulated in activated MuSCs (Le Grand et al., 2009). However, we could detect Vangl2 expression, although at a low level, in untreated mouse plantaris muscle, and Vangl2 was found to co-localize with M-cadherin at the MuSC/myofiber boundary (Figures 1A,B). N-cadherin is also thought to be expressed at the MuSC–myofiber boundary, as the scaffold protein for classical cadherins, β-catenin, has been localized to this region (Goel et al., 2017). We found a physical interaction between M-cadherin and Vangl2 in C2C12 cells. In contrast, we did not detect an interaction between N-cadherin and Vangl2, unlike a previous report using neurons (Nagaoka et al., 2014a). Considering that M-cadherin is expressed at substantially higher levels than N-cadherin in skeletal muscle tissue (Hollnagel et al., 2002; Valat et al., 2022) and that M-cadherin protein levels were also higher than those of N-cadherin in C2C12 cells (Figure 1C), Vangl2 may preferentially interact with M-cadherin in this cellular context. Nonetheless, the possibility that Vangl2 interacts with N-cadherin cannot be excluded, as the interaction may simply have been below our detection threshold. Moreover, the binding pattern of M-cadherin to Vangl2 was different from that of N- and E-cadherins. Specifically, Vangl2 did not compete with β-catenin for interaction with M-cadherin; rather, it formed a ternary complex with them (Figure 2G). To determine the binding pattern between cadherins and Vangl2, tertiary complex structure prediction was performed using AlphaFold3 (Supplementary Figure S2A). It should be noted that the predicted template modeling (pTM) score, which is an integrated measure of how efficiently the AlphaFold-multimer predicts the overall structure of the complex, was found to be moderate. However, the interface-predicted template modeling (ipTM) score, which measures the accuracy of the predicted relative positions of the subunits forming the protein-protein complex, was rather low in each prediction (Gomez and Kovalevskiy, 2025). However, structure of β-catenin binding region of cadherins indicated by blue in Supplementary Figure S2B and C was different among E−, N-, and M-cadherins (Huber and Weis, 2001). In summary, Vangl2 occupies the β-catenin interaction region of E− and N-cadherins, while this region in M-cadherin appears to be free. Thus, difference of this loop region in cadherins might affect the pattern of Vangl2/cadherin/β-catenin interaction. In addition, comparison of the predicted binding sites between Vangl2 and the cadherins revealed distinct residue interactions. The side chain of E389 in Vangl2 formed a hydrogen bond with R893 in N-cadherin and the distance between Cα atoms of these residues was close (6.65 Å). Although the homologous residue in E-cadherin, R870, was positioned close to E389 in Vangl2 (7.27 Å), the corresponding residue in M-cadherin, R773, was more distantly located (12.88 Å) (Supplementary Figure S2D). Meanwhile, hydrogen bonds were predicted between Q403 in Vangl2 and M780 in M-cadherin, as well as between T416 in Vangl2 and Y764 in M-cadherin and the distance between Cα atoms of these residues were close (6.14 Å and 5.54 Å respectively). In contrast, the distances of Cα atoms between Vangl2 residues and their homologous residues in E− and N-cadherin were more than twice those observed in M-cadherin (Q403 in Vangl2 and M877 in E-cadherin: 13.16 Å, Q403 in Vangl2 and M900 in N-cadherin: 16.85 Å, T416 in Vangl2 and Y861 in E-cadherin: 11.65 Å, T416 in Vangl2 and Y884 in N-cadherin: 19.33 Å) (Supplementary Figure S2E,F). These differences in residue interactions may influence the structural organization of the complexes.

Vangl2 knockdown inhibited C2C12 cell fusion (Figure 3A). This result was similar to the effect of M-cadherin knockdown in C2C12 cells (Charrasse et al., 2006; Wang et al., 2011). M-cadherin inhibits phosphorylation of β-catenin and suppresses GSK-3β activation (Wang et al., 2011; Wang et al., 2013). However, Vangl2 knockdown did not affect β-catenin phosphorylation and GSK-3β activation, although the PCP pathway attenuated the canonical Wnt/β-catenin signaling pathway (Yamamoto et al., 2008; Mentink et al., 2018). Meanwhile, it was reported that non-canonical Wnt signaling can activate the canonical Wnt/β-catenin signaling pathway depending on receptor context (Chen et al., 2000; Mikels and Nusse, 2006). MyoD, Myomaker, and Myomixer expression levels were positively regulated by the Wnt/β-catenin signaling pathway (Cui et al., 2019; Chen et al., 2020). Since we showed that Vangl2 knockdown downregulated the expression of MyoD and Myomixer (Figures 3L,M), it is possible that Vangl2 regulates β-catenin phosphorylation or stabilization. However, further investigation is required. On the other hand, we demonstrated that the cell surface expression levels of not only M-cadherin but also N-cadherin were reduced when Vangl2 was knocked down in C2C12 cells (Figure 4). Rab35 controls the endocytic and recycling pathways that regulate cell–cell junction localization of M- and N-cadherins. The knockdown of Rab35 reduces the cell surface expression of M- and N-cadherins and suppress the fusion of C2C12 cells (Charrasse et al., 2013). Overexpression of the E-cadherin cytoplasmic domain inhibits the cell surface expression of M- and N-cadherins and fusion of C2C12 cells (Ozawa, 2015). Thus, cell surface localization of cadherins in myoblasts is responsible for myogenic differentiation. It has been suggested that Vangl2 regulates cell surface expression of cadherins and is important for myogenic differentiation. Although an N-cadherin/Vangl2 interaction was not detected (Figure 1C), the surface expression of N-cadherin was synchronized with that of M-cadherin. The amino-terminal EC1 domain of E-cadherin governs both type-specific adhesive dimerization and lateral associations between cadherins (Chitaev and Troyanovsky, 1998). The lateral cadherin complex is transiently formed on the cell surface, serving as a source of cadherins available for participation in cell–cell adhesion. Lateral heterocomplexes can form between different classic cadherins (e.g., E− and P-cadherins) at the membrane (Klingelhöfer et al., 2000). Thus, a lateral interaction between M- and N-cadherin may be formed, maintaining N-cadherin at the membrane. Another possibility is a difference in cellular context. Endocytosis of N-cadherin depends on the small GTPase Rab5 which is known as early endosome marker in neuron (Kawauchi et al., 2010). However, both the expression and activation levels of Rab5a are much lower in undifferentiated C2C12 cells than in differentiated C2C12 cells (Cong et al., 2020). Therefore, under our experimental conditions, N-cadherin endocytosis may have been kept at low levels. We did not perform this experiment under differentiation conditions, but the behavior of N- and M-cadherins endocytosis may differ from that in undifferentiated cells. Additional endocytic or vesicle-associated proteins may contribute to the membrane localization of cadherins in C2C12 cells. Further investigation will be required.

In models of muscular dystrophy, such as Duchenne muscular dystrophy and limb-girdle muscular dystrophy-1C, altered expression of caveolin-3, a muscle membrane protein, leads to changes in M-cadherin levels. Overexpression of caveolin-3 downregulates M-cadherin and inhibits myoblast fusion, whereas its absence upregulates M-cadherin expression and enhances fusion. These findings directly link altered M-cadherin expression with the muscular dystrophy phenotype, suggesting that impaired myoblast fusion due to abnormal cadherin expression contributes to disease progression (Volonte et al., 2003). Stem cells expressing M-cadherin have been shown to restore dystrophin expression and improve muscle function in dystrophic muscles, further highlighting the importance of cadherin-mediated cell adhesion in muscle repair and regeneration (Torrente et al., 2004).

In conclusion, we revealed that Vangl2 interacts with M-cadherin and regulates myoblast fusion by stabilizing its cell surface expression. Further investigation of this interaction may contribute to skeletal muscle pathologies such as muscular dystrophy.

## Data Availability

The original contributions presented in the study are included in the article/Supplementary Material, further inquiries can be directed to the corresponding author.
