# Supplementary material for: Planar cell polarity protein Vangl2 interacts with M-cadherin and stabilizes its cell surface expression in mouse C2C12 myoblasts
Source: Front Cell Dev Biol. 2026 Jan 16;14:1701716. doi: 10.3389/fcell.2026.1701716 (PMC12856500; doi:10.3389/fcell.2026.1701716)
Supplement: Supplementary file 1 [file DataSheet1.docx]

Supplementary Material

**Contents**

Supplementary Materials and Methods

Supplementary Figure 1 Interaction among M-cadherin, β-catenin and Vangl2

Supplementary Figure 2 Structure prediction of cadherin/Vangl2 complex by AlphaFold3

Supplementary Figure 3 Myogenic gene expression analysis in Vangl2 knocked-down C2C12 cells

Supplementary Table 1 Antibodies used in this study

Supplementary Table 2 Oligonucleotides used to construct the plasmids in this study

Supplementary Table 3 Sequence of DsiRNAs used in this study

Supplementary Table 4 Sequences of primers for qPCR used in this study

**Supplementary Materials and Methods**

**Structure prediction by AlphaFold3**

Amino acid sequences of C-terminal domain of mouse Vangl2 (residues 240-521, Uniprot Accession No.: Q91ZD4), and cytoplasmic region of mouse E-cadherin (residues 734-884, Uniprot Accession No.: P09803), N-cadherin (residues 746-906, Uniprot Accession No.: P15116), and M-cadherin (residues 626-784, Uniprot Accession No.: P33146), respectively, are submitted to the AlphaFold3 server (https://alphafoldserver.com/) for processing and prediction of Vangl2/cadherin complexes (Abramson et al., 2024).





**Supplementary Figure 1 Interaction among M-cadherin, β-catenin and Vangl2.**

(A) Overexpressed M-cadherin was co-immunoprecipitated with endogenous β-catenin in 293T cells. M-cadherin-myc WT and S/D mutant were co-immunoprecipitated with β-catenin. Meanwhile, M-cadherin ΔCBD and S/A mutants lacked affinity for β-catenin. (B) The signal intensity of M-cadherin co-immunoprecipitated with β-catenin was normalized to the signal intensity of immunoprecipitated β-catenin and plotted (n = 3). One-way ANOVA: P < 0.0001. Tukey’s multiple comparisons test: *; P < 0.05, **; P < 0.005, ****; P < 0.0001. (C) β-catenin-HA was co-expressed with M-cadherin and Vangl2 in 293T cells. Each cell lysate was immunoprecipitated by anti-myc antibodies. (D) Band intensity of co-immunoprecipitated FLAG-Vangl2 relative to the intensity of immunoprecipitated M-cadherin-myc was plotted (n = 3). Welch’s *t*-test: *; P < 0.05. (E) Band intensity of co-immunoprecipitated β-catenin-HA relative to the intensity of immunoprecipitated M-cadherin-myc was plotted (n = 3). Welch’s *t*-test: P = 0.45.


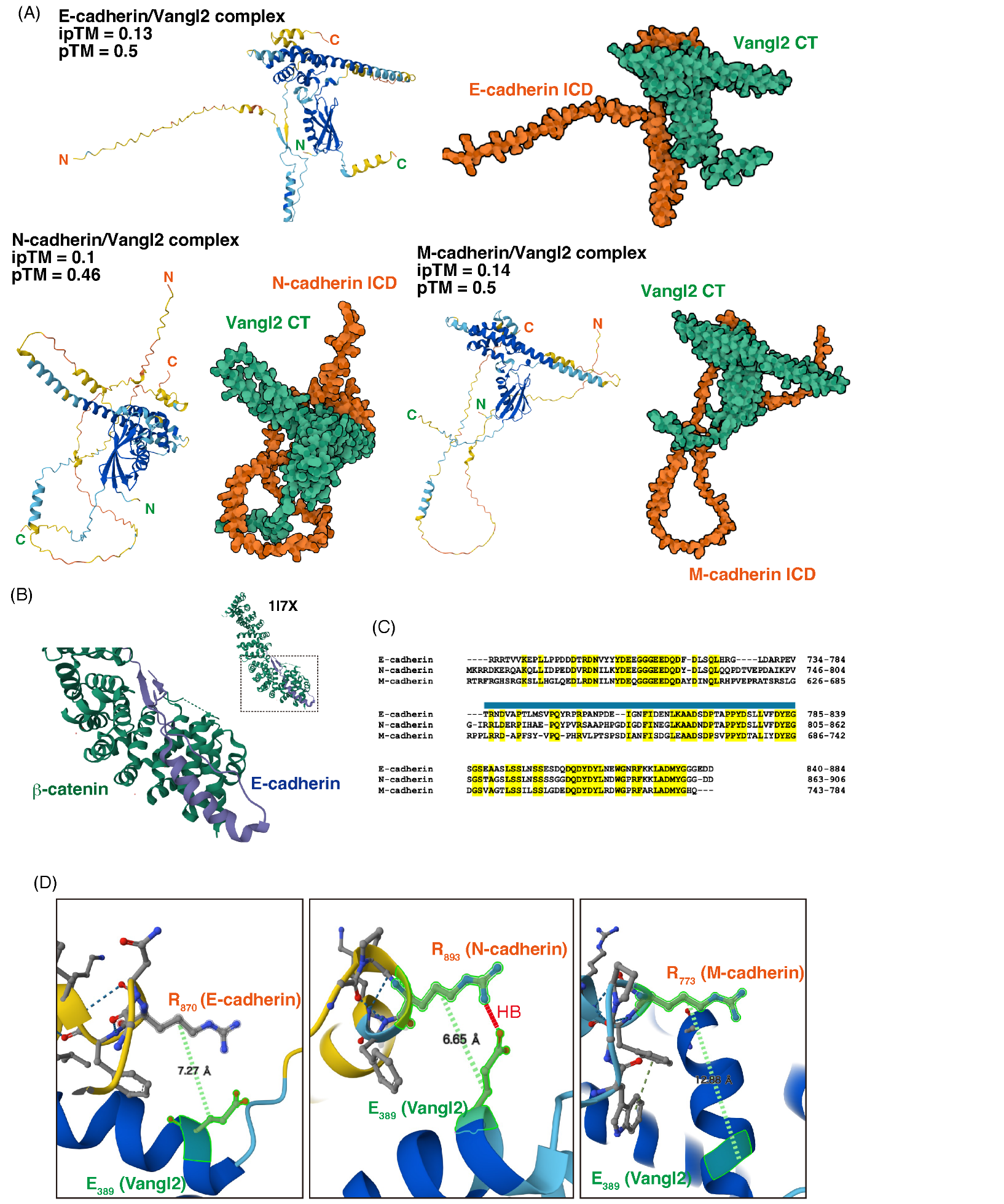


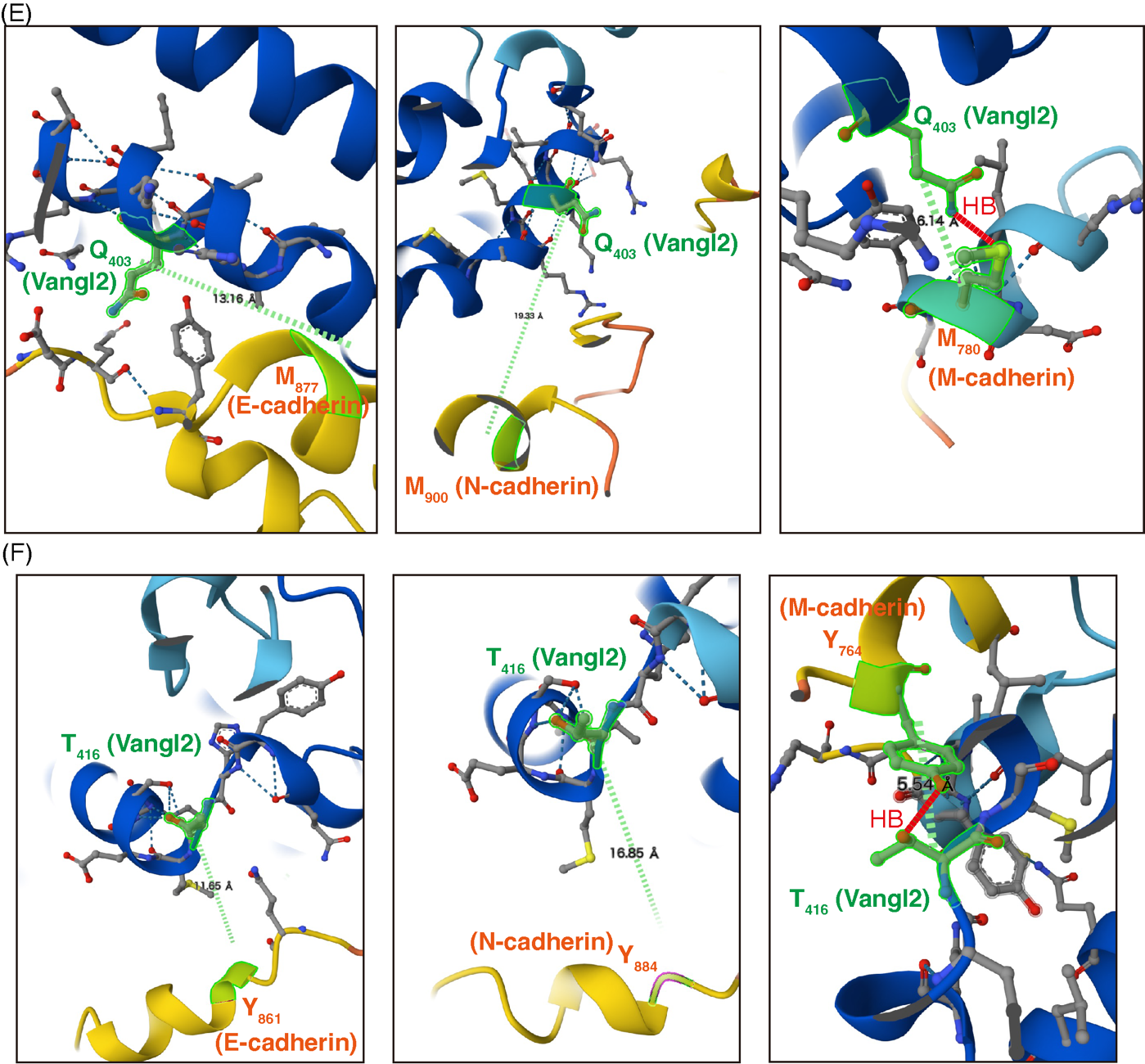


**Supplementary Figure 2 Structure prediction of cadherin/Vangl2 complex by AlphaFold3**

(A) ICD of cadherins and Vangl2 CT complex structure prediction. The left panel of each prediction shows the ribbon diagram. The ribbon colors were divided into four levels depending on the predicted local distance difference test (plDDT) level (blue: very high; plDDT > 90, cyan: confident; 90 > plDDT > 70, yellow: low; 70 > plDDT > 50, orange: very low; plDDT < 50). The right panel shows cadherin and Vangl2 protein chains in orange and green colors, respectively. (B) Partial crystal structure of E-cadherin/β-catenin complex (PDB ID: 1I7X) (Huber & Weis, 2001). The area surrounded by dotted box in upper right full structure is magnified. The blue and green ribbons indicate E-cadherin and β-catenin chains, respectively. (C) Amino acid sequence alignment comparison of the ICDs of E-cadherin, N-cadherin, and M-cadherin. The blue line in panel (B) indicates the region of E-cadherin interacting with β-catenin. Conserved amino acids among E-cadherin, N-cadherin, and M-cadherin are highlighted in yellow. (D - F) Residues which hydrogen bond between Vangl2 and cadherin proteins (HB) were predicted. HBs were indicated by broken red line. Distance was also estimated between Cα atoms of indicated residues and indicated by broken light green line.





**Supplementary Figure 3 Myogenic gene expression analysis in *Vangl2* knocked-down C2C12 cells.**

DsiRNAs were transfected into C2C12 cells, which were then incubated for 48 h. Total RNA was isolated from the cells, and reverse transcription was performed to obtain cDNA. Gene expression was evaluated using qPCR and plotted (n = 3). One-way ANOVA: *Pax7*; P = 0.420, *Myf5*; P = 0.285, *Myogenin*; P = 0.156, *Cdh15*; P = 0.644.

**Supplementary Table 1 Antibodies used in this study**

| Antigen | Host | Supplier | Clone ID or product number | Application (Dilution) | Conjugation |
| --- | --- | --- | --- | --- | --- |
| α-Tubulin | Mouse | Proteintech | 1E4C11 | WB (1:1000) |  |
| Active β-catenin | Mouse | Sigma | 8E7 | WB (1:1000) |  |
| Akt (pan) | Rabbit | CST | C6E7 | WB (1:1000) |  |
| β-catenin | Mouse | BD | 14/Beta-Catenin | IP (1:300)  WB (1:2000) |  |
| c-Jun | Rabbit | CST | 60A8 | WB (1:1000) |  |
| Connexin 43 | Rabbit | CST | #3512 | WB (1:1000) |  |
| FLAG | Mouse | Wako | 1E6 | IP (1:600)  WB (1:2000) |  |
| GAPDH | Rabbit | CST | 14C10 | WB (1:1000) |  |
| GSK-3β | Rabbit | GeneTex | GTX111230 | WB (1:1000) |  |
| HA | Rat | Roche | 3F10 | IP (1:300)  WB (1:1000) |  |

**Supplementary Table 1 (Continued) Antibodies used in this study**

| Antigen | Host | Supplier | Clone ID or product number | Application (Dilution) | Conjugation |
| --- | --- | --- | --- | --- | --- |
| Laminin | Rabbit | Sigma | L9393 | IF (1:1000) |  |
| M-cadherin | Rabbit | CST | D4B9L | WB (1:2000) |  |
| M-cadherin | Sheep | R&D | AF7677 | IF (1:500) |  |
| Mouse IgG | Donkey | Thermo | A10037 | IF (1:1000) | Alexa 568 |
| Mouse IgG | Goat | Biorad | 170-6516 | WB (1:5000) | HRP |
| Mouse IgG light chain specific | Goat | Jackson | 115-035-174 | WB (1:5000) | HRP |
| Myc | Mouse | MBL | My3 | IP (1:600)  WB (1:2000) |  |
| MyHC | Mouse | DSHB | MF20 | IF (1:50) |  |
| MYPT1 | Rabbit | CST | D6C1 | WB (1:1000) |  |
| N-cadherin | Mouse | BD | 32/N-Cadherin | WB (1:2000) |  |
| N/A | Mouse | BD | 557273 | IP (1:50) |  |
| Pax7 | Mouse | SCBT | PAX7 | WB (1:500) |  |
| Phospho Akt (Ser473) | Rabbit | CST | D9E | WB (1:1000) |  |

**Supplementary Table 1 (Continued) Antibodies used in this study**

| Antigen | Host | Supplier | | Clone ID or product number | | Application (Dilution) | Conjugation |
| --- | --- | --- | --- | --- | --- | --- | --- |
| Phospho c-Jun (Ser63) | Rabbit | | CST | | E6I7P | WB (1:1000) |  |
| Phospho GSK-3β (Ser 9) | Rabbit | | CST | | D85E12 | WB (1:1000) |  |
| Phospho MYPT1 (Thr853) | Rabbit | | CST | | 4563 | WB (1:1000) |  |
| Rabbit IgG | Donkey | | Abcam | | AB150063 | IF (1:1000) | Alexa 647 |
| Rabbit IgG light chain specific | Goat | | Jackson | | 211-032-171 | WB (1:5000) | HRP |
| Rat IgG | Donkey | | Abcam | | AB150153 | IF (1:1000) | Alexa 488 |
| Rat IgG | Goat | | SCBT | | sc-2030 | WB (1:5000) | HRP |
| Sheep IgG | Donkey | | Thermo | | A21099 | IF (1:1000) | Alexa 568 |
| Vangl2 | Mouse | | SCBT | | C-2 | IP (1:50) |  |
| VANGL2 | Rat | | Millipore | | 2G4 | IF (1:100)  WB (1:500) |  |

N/A: Not applicable, Abcam; Abcam (Cambridge, UK), BD; BD biosciences (Franklin Lakes, NY), Biorad; Bio-rad (Hercules, CA), CST; Cell Signaling Technology (Danvers, MT), DSHB; Developmental Studies Hybridoma Bank (Iowa City, IA), GeneTex; Gene Tex (Irvine, CA), Jackson; Jackson ImmunoResearch (West Grove, PA), MBL; Medical and Biological Laboratories (Tokyo, Japan), Proteintech; Proteintech (Rosemont, IL), R&D; R&D systems (Minneapolis, MN), Roche; Roche (Basel, Switzerland), SCBT; Santa Cruz Biotechnology (Dallas, TX), Sigma; Sigma-Aldrich (St. Louis, MO), Thermo; Thermo Fisher Scientific, Wako; FUJIFILM Wako Pure Chemical (Osaka, Japan), IF; immunofluorescence, IP; immunoprecipitation, WB; western blotting.

**Supplementary Table 2 Oligonucleotides used to construct the plasmids in this study**

| Name | Sequence (5’->3’) |
| --- | --- |
| M-cadherin Fw | AAAGCTTACCATGGGTTCTGCTCTGCTCCT |
| M-cadherin Re | TCTCACTGATGTCCATACATGTCC |
| M-cadherin ΔJMDC Fw | TAACTATCCAGTGGAGCCGAGGGCCA |
| M-cadherin ΔJMDC Re | TCCACTGGATAGTTAAGGATGTTGTCCCGAAGGTC |
| M-cadherin ΔCBD Fw | GTGATGGCGGACATCAGAATTCCGAGCAAA |
| M-cadherin ΔCBD Re | GATGTCCGCCATCACTGATGAAGTTGGCA |
| M-cadherin S/A Fw | GGCCGCCATTCTGGCCGCCCTGGGAGATGAAGACCAGGACT |
| M-cadherin S/A Re | GCCAGAATGGCGGCCAGCGTCCCTGCCACAGCGCCAT |
| M-cadherin S/D Fw | GGACGACATTCTGGACGACCTGGCGGACATGTATGGA |
| M-cadherin S/D Re | TCCAGAATGTCGTCCAGCGTCCCTGCCACATCGCCAT |
| 3x myc tag S | AATTCCGAGCAAAAGCTCATCTCAGAGGAAGATCTGGAATTA  GAGCAAAAGCTCATCTCAGAGGAAGATCTGGAATTAGAGCA  AAAGCTCATCTCAGAGGAAGATCTGTGAT |

**Supplementary Table 2 (Continued) Oligonucleotides used to construct the plasmids in this study**

| Name | Sequence (5’->3’) |
| --- | --- |
| 3x myc tag AS | AATTCAGATCTTCCTCTGAGATGAGCTTTTGCTCTAATTCCAG  ATCTTCCTCTGAGATGAGCTTTTGCTCTAATTCCAGATCTTCC  TCTGAGATGAGCTTTTGCTCCATGGTAT |
| β-catenin Fw | ACCGCTGCTAGCACAATGGCTACTCAAGCTGACC |
| β-catenin Re | GGACGGGATCCCAGGTCAGTATCAAACCAGGCC |
| 3x HA tag S | GATCCTACCCATATGACGTTCCAGACTACGCGTATCCGTACGA  CGTTCCGGATTACGCTTACCCTTACGACGTACCTGACTACGCT  TAAGC |
| 3x HA tag AS | GGCCGCTTAAGCGTAGTCAGGTACGTCGTAAGGGTAAGCGTA  ATCCGGAACGTCGTACGGATACGCGTAGTCTGGAACGTCATAT  GGGTAG |
| Human VANGL2 Fw | AAGCTTACCATGGACACCGAGTCCCAGT |
| Human VANGL2 Re | TCTAGATCACACTGAGGTCTCAGACT |
| Human VANGL2 ΔCT Fw | GCTGGAGTGATCTAGAGGGCCCGTTTA |
| Human VANGL2 ΔCT Re | CTAGATCACTCCAGCAGGACCACGGCCA |

Abbreviations: Fw; forward, Re; reverse, S; sense, AS; antisense

**Supplementary Table 3 Sequence of DsiRNAs used in this study**

| Name | Product Name | Duplex Sequence |
| --- | --- | --- |
| NC siRNA | DS NC-1 | N/A |
| Vangl2 siRNA#1 | mm.Ri.Vangl2.13.1 | 5’-ACUUCAGCUUGGUAGUGAGCACCAA-3’  5’-UUGGUGCUCACUACCAAGCUGAAGUCC-3’ |
| Vangl2 siRNA#2 | mm.Ri.Vangl2.13.2 | 5’-CAGUUUGCCGUUUCUCUAGUGGATG-3’  5’-CAUCCACUAGAGAAACGGCAAACUGAA-3’ |

N/A: Not available

**Supplementary Table 4 Sequences of primers for qPCR used in this study**

| **Gene** | **Forward (5’ -> 3’)** | **Reverse (5’ -> 3’)** |
| --- | --- | --- |
| *36B4* (Rplp0) | TGTTTGACAACGGCAGCATTT | CCGAGGCAACAGTTGGGTA |
| *Cdh15*  (M-cadherin) | CATCCCACCCATTAGTGTGTC | TCCCAGTGAACTTGTCGATAGA |
| *Myf5* | AAGGCTCCTGTATCCCCTCAC | TGACCTTCTTCAGGCGTCTAC |
| *Myogenin* | GAGACATCCCCCTATTTCTACCA | GCTCAGTCCGCTCATAGCC |
| *Myomaker* | ATCGCTACCAAGAGGCGTT | CACAGCACAGACAAACCAGG |
| *Myomixer* | GTTAGAACTGGTGAGCAGGAG | CCATCGGGAGCAATGGAA |
| *MyoD* | CCACTCCGGGACATAGACTTG | AAAAGCGCAGGTCTGGTGAG |
| *Pax7* | TCTCCAAGATTCTGTGCCGAT | CGGGGTTCTCTCTCTTATACTCC |

**Reference**

Abramson, J., Adler, J., Dunger, J., Evans, R., Green, T., Pritzel, A., Ronneberger, O., Willmore, L., Ballard, A. J., Bambrick, J., Bodenstein, S. W., Evans, D. A., Hung, C. C., O'Neill, M., Reiman, D., Tunyasuvunakool, K., Wu, Z., Žemgulytė, A., Arvaniti, E., Beattie, C., Bertolli, O., Bridgland, A., Cherepanov, A., Congreve, M., Cowen-Rivers, A. I., Cowie, A., Figurnov, M., Fuchs, F. B., Gladman, H., Jain, R., Khan, Y. A., Low, C. M. R., Perlin, K., Potapenko, A., Savy, P., Singh, S., Stecula, A., Thillaisundaram, A., Tong, C., Yakneen, S., Zhong, E. D., Zielinski, M., Žídek, A., Bapst, V., Kohli, P., Jaderberg, M., Hassabis, D. and Jumper, J. M. (2024) Accurate structure prediction of biomolecular interactions with AlphaFold 3. Nature, 630(8016), 493-500.

Huber, A. H. and Weis, W. I. (2001) The structure of the beta-catenin/E-cadherin complex and the molecular basis of diverse ligand recognition by beta-catenin. Cell, 105(3), 391-402.
